# Development of Antimicrobial Biocomposite Films to Preserve the Quality of Bread

**DOI:** 10.3390/molecules23010212

**Published:** 2018-01-19

**Authors:** Kelly J. Figueroa-Lopez, Margarita María Andrade-Mahecha, Olga Lucía Torres-Vargas

**Affiliations:** 1 Optoelectronics Group, Interdisciplinary Science Institute, Faculty of Basic Science and Technologies, Universidad del Quindío, Carrera 15 Calle 12 Norte, Armenia 630004, Colombia; kjfigueroal@iata.csic.es; 2 Group of Research on Agroindustrial Processes (GIPA), Universidad Nacional de Colombia, Palmira 763533, Colombia; 3 Group of Agro-industrial Sciences, Faculty of Agro-industrial Sciences, Universidad del Quindío, Carrera 15 Calle 12 Norte, Armenia 630004, Colombia; oltorres@uniquindio.edu.co

**Keywords:** biocomposite films, gelatin, oleoresins, antimicrobial compounds, food quality

## Abstract

This study focused on the development of gelatin-based films with incorporation of microcrystalline cellulose as reinforcement material. Clove (*Syzygium aromaticum*), nutmeg (*Myristica fragrans*), and black pepper (*Piper nigrum*) oleoresins containing antimicrobial compounds of natural origin were incorporated into the films. The mechanical, thermal, optical, and structural properties, as well as color, seal strength and permeability to water vapor, light, and oil of the films were determined. Adding oleoresins to the gelatin matrix increased the elongation of the material and significantly diminished its permeability to water vapor and oil. Evaluation of the potential use of films containing different oleoresins as bread packaging material was influenced by the film properties. The biocomposite film containing oleoresin from black pepper was the most effective packaging material for maintaining bread’s quality characteristics.

## 1. Introduction

Biocomposite materials are structures made up of at least one phase (matrix or reinforcement). One of these phases is obtained from a renewable source [1,2]. Proteins, polysaccharides, or a mixture of these which can provide mechanical and/or barrier properties, are some of those commonly used to make up the polymer structure [3,4]. Gelatin is one of the proteins with greatest industrial application due to its low melting point; in addition, it has a unique amino acid sequence, with high contents of proline, glycine, and hydroxyproline, which help in the formation of flexible films [5]. Gelatin is a good barrier against oxygen and carbon dioxide, but a poor barrier against water vapor [6]. To obtain materials with better flexibility, manageability, and extension ability, it is necessary to incorporate substances of low molecular weight, like plasticizers [7]. Also, compounds with antimicrobial and antioxidant activity can be incorporated, which help to improve the physical-chemical and microbiological properties of polymer materials and may improve the quality of the food they cover and/or increase its shelf life [8]. Different lipids (oleoresins and essential oils) have shown antimicrobial or antioxidant properties, which may be used for the development of biocomposite materials. Lipids are a good barrier against water vapor. Additionally, they may decrease film transparency, which is important to preserve the quality of food exposed to light [9,10]. Thus, the formation of active biocomposite materials consists of incorporating compounds with antioxidant or antimicrobial properties to the polymer matrix, which permit, through the migration of the active compounds onto the food surface, the conservation and extension of its shelf life [11,12]. Nevertheless, film properties are mainly influenced by the polymers used, active compounds, and preparation method. The use of biocomposite materials as food packaging or wrapping will depend on the mechanical properties and the permeability to water vapor [13]. The aim of this work was to investigate the effect of adding natural antimicrobial compounds (clove, nutmeg, and black pepper oleoresins) incorporated into gelatin based biocomposite films, on their moisture content, water vapor permeability, oil permeability, tensile properties, structure, optical and thermal properties. Biocomposite films were evaluated as packaging material on bread slices stored at ambient conditions (around 25 °C and 75% relative humidity). The physical-chemical, microbiology, and sensory characteristics of bread were evaluated for nine days. 

## 2. Materials and Methods

### 2.1. Materials 

To prepare the films, type B food-grade gelatin was used with a bloom of 220–240 g (Gelco S.A., Barranquilla, Colombia), along with pharmaceutical-grade microcrystalline cellulose, Avicel^®^ PC 105 (FMC Biopolymer, Campinas, Brazil); 99% purity glycerol (Sigma Aldrich, St. Louis, MO, USA); Tween 80 for synthesis (Merck, Darmstadt, Germany); and clove, nutmeg, and black pepper oleoresins (TECNAS S.A., Antioquia, Colombia).

### 2.2. Minimum Inhibitory Concentration of the Oleoresins

The minimum inhibitory concentration (MIC) of the oleoresins against *S. aureus* and *E. coli* was determined by using the classic plate micro-dilution method by the National Committee of Laboratory Safety and Standards (NCLSS) [14]. Triplicate experiments were performed for each concentration of natural antimicrobials (4%, 2%, 1%, 0.5%, and 0.25%). The lowest concentration of the natural antimicrobial that showed growth inhibition was considered the MIC. 

### 2.3. Physical-Chemical Analysis

Gelatin and microcrystalline cellulose (MCC) particle size were determined by using a Zetasizer nano ZS90 (Malvern Instruments, Ltd., Malvern, UK). Gelatin and MCC crystallinity were determined by using an X-ray diffractometer (Siemens, Karlsruhe, Germany) equipped with a Cu anode tube (which emits radiation of λ = 0.154 nm) with a voltage of 40 kV and current of 30 mA. The diffractograms were analyzed by using the Origin 8 program and crystallinity (% $Ic_{r}$) was determined by using Equation (1):

(1) $$Ic_{r}=\frac{I_{200}-I_{non-cr}}{I_{200}}\times 100$$

where *I*_200_: Maximum intensity value of the crystalline peak, *I_non-cr_*: Intensity value that separates both diffraction peaks

Identification of the principal functional groups of the MCC and the gelatin was made through Fourier transformed infrared spectroscopy (Perkin Elmer, Waltham, MA, USA). The spectra were obtained within a range from 4000 to 400 cm^−1^ and analyzed by using the Origin 8 program. Thermal characterization of the MCC, the gelatin, and the oleoresins (nutmeg, black pepper, and clove) was carried out through differential scanning calorimetry (DSC 214 Polyma, Netzsch, Selb, Germany); scanning was from −20 to 140 °C at a rate of 5 °C/min.

The chemical composition of the oleoresins (nutmeg, clove, and black pepper) was determined by using a gas chromatograph AT 6890 Series Plus (Agilent Technologies, Palo Alto, CA, USA), coupled to a selective mass detector (Agilent Technologies, MSD 5973) operating in full scan mode. The column used in the analysis was DB-5MS (5%-phenylpoly(methylsiloxane), 60 m × 0.25 mm × 0.25 µm) and the injection was conducted in split mode (30:1) with Viny = 2 µL.

### 2.4. Preparation of Films 

Films were prepared through the “casting” method following the methodology proposed by Andrade-Mahecha et al. [15] and by Mondragón et al. [16] with some modifications. The film-forming solution (FFS) was obtained from an aqueous suspension of microcrystalline cellulose (0.15 g/100 g of FFS), which was magnetically agitated for 30 min at 35 °C. Simultaneously, two aqueous suspensions were also prepared; one containing gelatin (3 g/100 g of FFS) stirred at 60 °C for 30 min and another containing glycerol (0.45 g/100 g of FFS) stirred at 35 °C for 15 min. Thereafter, all the components were mixed and kept at 60 °C for 15 min under constant stirring. Next, the respective oleoresin containing the natural antimicrobial compounds (10 g (500 µL of oleoresin/500 µL Tween 80 at 2%)/100 g FFS) was added. The FFS was kept at 45 °C for 15 min, prior to being poured into nonstick molds to be subjected to convection drying (45 °C during 4 h) on a stove with forced-air circulation (FD-53, Binder, Tuttlingen, Germany). 

### 2.5. Film Characterization

Film thickness was measured at five different points by using a digital micrometer (Mitutoyo, Corp., Kawasaki, Japan).

#### 2.5.1. Humidity Content

Film humidity content was determined immediately after drying and after a conditioning period (58% RH during 48 h), according to the gravimetric method by ASTM D644-99 [17].

#### 2.5.2. Water Vapor Permeability

Water vapor permeability (WVP) was determined by following the ASTM E96-05 [18]. The films were placed on stainless steel cells subjected to external environments with different relative humidity (RH 2–33%, 33–66%, and 64–90%). Weight loss of the cells was monitored every hour for 9 h.

#### 2.5.3. Mechanical Properties

Mechanical tests were carried out on a texture analyzer (TA-XT plus, Stable Micro Systems, Surrey, UK), at an operating rate of 1 mm/s and separation of 40 mm between clamps. The mechanical properties evaluated were: tensile strength (MPa), elongation (%), and young’s modulus (MPa), according to ASTM D882-02 [19].

#### 2.5.4. Oil Permeability 

Oil Permeability was determined by following the methodology proposed by Yan et al. (2012) [20], using the following equation:

$$\mathrm{Po}=\frac{\Delta W\times \mathrm{FT}}{A\times T}$$

where ∆W is the weight variation of the filter paper (g), FT is the film thickness (mm), A is the area of effective contact (m^2^), and T is the storage period (days).

#### 2.5.5. Measurement of Seal Strength

Film samples (7.62 × 2.54 cm) were thermally sealed for two seconds, one on top of another with an area of 2.54 cm × 0.2 cm, using a manual thrust sealer (Hongzhan, KS-100, Wenzhou, China). Seal strength was determined through the ASTM F-88 [21], using a texture analyzer (TA-XT plus, Stable Micro Systems). Seal strength was calculated with the maximum force on the film width [22]. 

#### 2.5.6. X-ray Diffraction (XRD)

Film samples (4 × 4 cm) were analyzed in an X-ray diffractometer (Bruker D8-Advance, Rheinstetten, Germany) equipped with a tube with Cu anode (radiation of λ = 0.154 nm) with voltage of 30 Kv, current of 35 Ma, filter for the K_β_ line, and a scintillation detector. The diffractograms were analyzed by using the Origin 8 program.

#### 2.5.7. Fourier Transform Infrared (FTIR) Spectroscopy 

Identification of the principal functional groups was conducted via spectroscopy equipment (IR Prestige 21 Shimadzu, Kyoto, Japan). Spectra were obtained within a wave number range of 4000 to 400 cm^−1^ and analyzed by using the Origin 8 program. 

#### 2.5.8. UV-Vis Light Transmittance Values

Film light barrier (50 × 30 mm samples) was evaluated in wavelengths from 200 to 800 nm in a spectrophotometer (Thermo Scientific Genesys-10S-UV-Vis, Waltham, MA, USA) according to Leceta et al. [23]. 

#### 2.5.9. Color

Film color was determined by using a spectrocolorimeter (Hunter Associates Laboratory, Inc., Reston, VA, USA), according to Arfat et al. [13]. The color difference (∆E*) was calculated by using the following equation:

$$\Delta E^{*}={\left[{\left(\Delta L^{*}\right)}^{2}+{\left(\Delta a^{*}\right)}^{2}+{\left(\Delta b^{*}\right)}^{2}\right]}^{0.5}$$

where ∆E*, ∆a* and ∆b* corresponded to the differences between the color parameters of films containing oleoresins and the values of the reference film (without oleoresin) (L* = 2.094, a* = −1.46, b* = 4.73).

#### 2.5.10. Thermogravimetric Analysis (TGA)

Non-isometric degradation measurements were conducted on a Thermo Microbalance (TG 209 F1 Iris, Netzsch, Selb, Germany). Tests were run from 25 °C to 600 °C at a heating rate of 10 °C/min in a nitrogen atmosphere (10 mL/min) [23].

### 2.6. Influence of Biocomposite Films on Bread Quality

The formulation of the artisan bread was carried out according to Colombian standard NTC 1363 [24]. Two lots of bread manufactured on different days were evaluated. In both lots, bread samples were packaged in reference films (without oleoresin) and with oleoresins (clove, nutmeg, and black pepper). The bread samples (8 cm × 8 cm) were wrapped with two sheets of the biocomposite material (10 × 10 cm), which were thermally sealed on four sides. The physical-chemical stability (humidity, pH, aw, and weight loss) was evaluated on days 1, 3, 5, and 9 of the storage period at 25 °C and 75% RH. The microbiological and sensory quality of the bread was evaluated at days 1, 4, and 9.

### 2.7. Physical-Chemical Evaluation of the Bread

Humidity was determined by using the Colombian standard NTC 282 [25]. Five-gram amounts of the sample were taken in a metallic capsule, which was introduced with the samples into a stove with forced-air circulation (FD-53, Binder) between 100 and 110 °C and their weight was evaluated until constant weight. The pH was determined through NTC 1363. Water activity was determined by using a portable PawKit water activity (aw) meter (Aqualab, Pullman, WA, USA). Weight loss was calculated as the difference between the initial weight and the final weight of the bread sample. 

### 2.8. Microbiological Evaluation of the Bread

A microbiological count was conducted of *Escherichia coli* [26], *Staphylococcus aureus* positive coagulase [27], *Bacillus cereus* [28], along with detection of *Salmonella* spp. [29], mold and yeast [30], according to that indicated in the technical standard for bread.

### 2.9. Sensory Evaluation of the Bread

A descriptive sensory analysis was conducted with 10 untrained panelists following the NTC 3925 [31]. Attributes of aroma, flavor, hardness, and general acceptability were evaluated in a scale from 1 to 5. 

### 2.10. Statistical Analysis 

Each treatment was done in triplicate and the results of the properties were evaluated with 5% significance level (*p* ≤ 0.05). Analysis of variance (ANOVA) and a multiple comparison test (Tukey) permitted identifying significant differences among the treatments. The results were analyzed through the SPSS Statistics 22.0 software for Windows (SPSS Statistical software, Inc., Chicago, IL, USA). 

## 3. Results and Discussion

### 3.1. Physical-Chemical and Microbiological Study of the Components of the Biocomposite Material

Black pepper showed antimicrobial activity against both bacterial strains, starting at 0.5% (0.5 µL/100 µL), while clove started at 1% (1 µL/100 µL) against *Staphylococcus aureus* and as of 2% (2 µL/100 µL) against *Escherichia coli*. Likewise, nutmeg inhibited the growth of *Staphylococcus aureus* as of 0.5% (0.5 µL/100 µL) and as of 1% (1 µL/100 µL) against *Escherichia coli*. 

The type B gelatin used in this research had a Bloom of 220–240 g, which is related to the gel’s mechanical elasticity, an important parameter in its capacity and gelling force, for provoking deformation at a certain concentration and temperature. 

**Figure 1:**
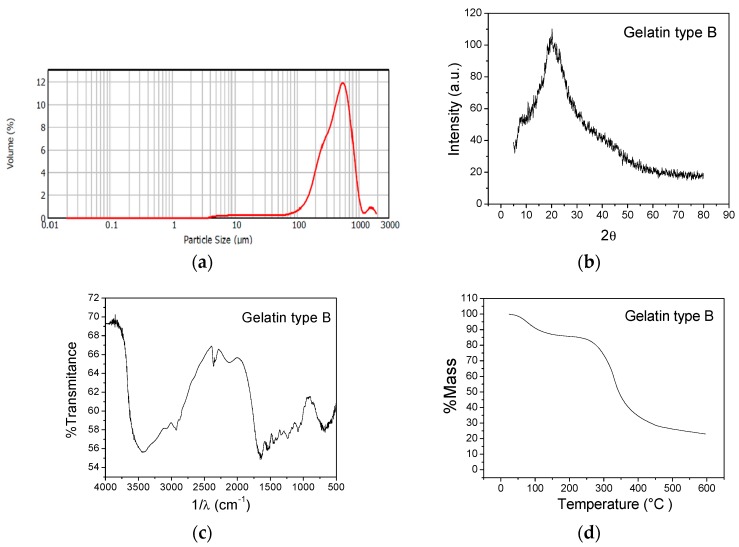
Structural and thermal characterization of type B gelatin. (**a**) Particle size distribution; (**b**) X-ray diffractogram; (**c**) FTIR spectrum; and (**d**) TGA thermogram.

Figure 1a shows the gelatin’s particle size distribution, which had unimodal distribution with a broad range of sizes comprised between 3.8 and 1905 µm. A large peak was observed where there are most of the particles with approximate sizes of 549.5 µm with 10.58% volume, this particle diameter is close to the mean diameter (D(4.3) = 476.987 µm).

Figure 1b displays the gelatin’s X-ray diffractogram, showing a dominant peak amply widened at 20.08°, which is characteristic of amorphous materials, indicating that gelatin does not have a periodic structure, given that it comprised of proteins with different structures [32].

Figure 1c shows the gelatin’s FTIR spectrum. The type A amide band at 3425 cm^−1^ corresponds to stretching of N-H groups with hydrogen bonds; at 2928 cm^−1^ there are type B amide bands that correspond to stretching of CH_2_; the band at 1638 cm^−1^ is attributed to type I amides; at 1543 cm^−1^, we find the type II amides and at 1232 cm^−1^ the type III amides [33,34].

Figure 1d shows three thermal degradation stages. The first stage at 191 °C with 14.15% mass loss, corresponding to water elimination; the second stage at 434 °C has the highest mass loss (55.55%), which was associated to degradation of proteins of high molecular weight and a residual mass (ashes) of 22.97%.

**Figure 2:**
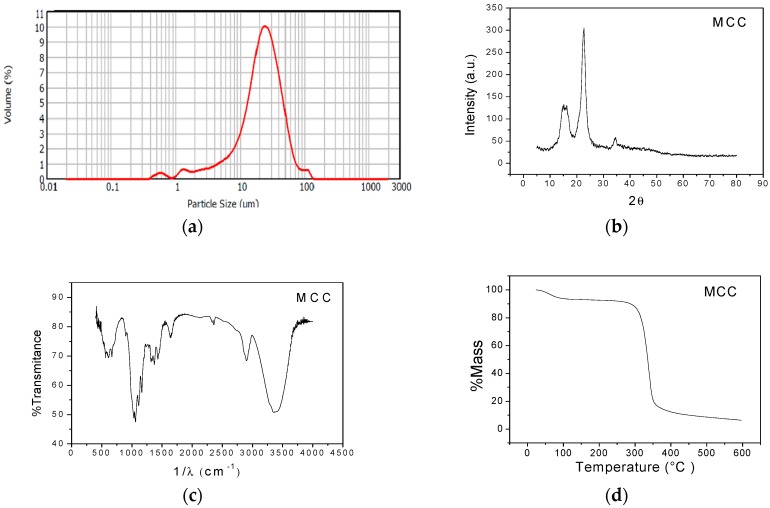
Structural and thermal characterization of MCC. (**a**) Particle size distribution; (**b**) X-ray diffractogram; (**c**) FTIR spectrum; and (**d**) TGA thermogram.

Figure 2a illustrates a unimodal distribution with size range between 0.41 and 138 µm. A large peak was observed where there is the majority of particles with approximate sizes of 26.3 µm with 9.06% volume; this particle diameter was close to the mean diameter (D(4,3) = 25.9 µm).

Figure 2b presents the microcrystalline cellulose diffractogram, with a peak in the 2θ angle at 22.59°, corresponding to the crystalline and a peak on the 2θ angle at 18.8°, corresponding to the amorphous material. The microcrystalline cellulose had 79.87% crystallinity, which coincides with the values reported in other studies [35,36,37].

Figure 2c shows the principal functional groups of the microcrystalline cellulose. At 3399 cm^−1^, stretching of the -OH groups was observed; the CH and CH_2_ groups are found at 2900 cm^−1^; the band at 1427 cm^−1^ is associated to the presence of HCH groups and OCH vibrations; the band at 1374 cm^−1^ is related to the vibration of CH groups; the band at 1217 cm^−1^ is attributed to C-C groups and at 1039 cm^−1^ to stretching of C-O groups [38,39].

Figure 2d shows that the first stage of thermal degradation corresponds to water elimination at 108 °C. Dehydration, decarboxylation, depolymerization, and glycosyl decomposition take place in the second stage between 363 and 589 °C [37,40,41].

The main active compounds identified in the nutmeg oleoresin were: myristic acid, miristicine, elemicine, sabinene and α-pinene. In the clove oleoresin, eugenol and eugenol acetate were identified. The black pepper oleoresin presented piperine as its main component, followed by *trans*-β-cariophylene and limonene. All these compounds have antimicrobial and antioxidant properties, as reported by other authors [42,43,44].

### 3.2. Characterization of Films

To obtain the films, 0.22 g of solution per each cm^2^ of support plate permitted obtaining a continuous cohesive matrix with thicknesses of 0.058 ± 0.0013 mm containing clove oleoresin, 0.06 ± 0.0021 mm containing nutmeg oleoresin, and 0.062 ± 0.0018 mm containing black pepper oleoresin.

#### 3.2.1. Humidity

**Figure 3:**
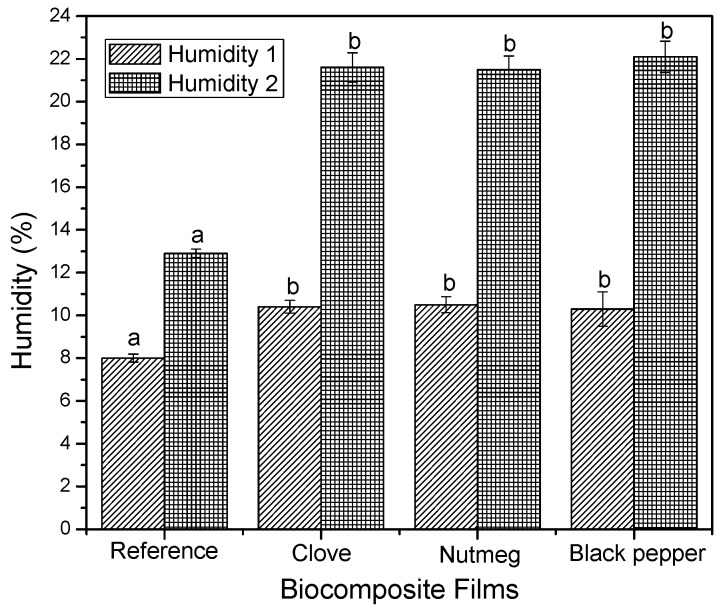
Humidity content of biocomposite films with oleoresins and reference before and after conditioning. Different letters in the same column indicate significant differences (*p* < 0.05).

Figure 3 presents the humidity of films after drying (Humidity 1) and after the conditioning period (Humidity 2). The humidity content of the biocomposite films containing oleoresins did not have significant differences after drying, obtaining humidity values of 10.4 ± 0.1 g of water/100 g of biocomposite film. After conditioning, the films increased their humidity content to 21.73 ± 0.32 g of water/100 g of biocomposite film. Additionally, the reference films (biocomposites without added oleoresin) had humidity contents of 8 ± 0.18 g of water/100 g of biocomposed film after drying and 12.9 ± 0.19 g of water/100 g of biocomposite film upon completing the conditioning period. The reference films had low humidity after drying and after conditioning with respect to the biocomposite films with oleoresins presenting significant differences (*p* < 0.05), which indicates that oleoresins would be creating bonds with the OH groups of the plasticizer and its polyphenolic groups, thus, maintaining the structure hydrated [45]. 

#### 3.2.2. Water Vapor Permeability

**Figure 4:**
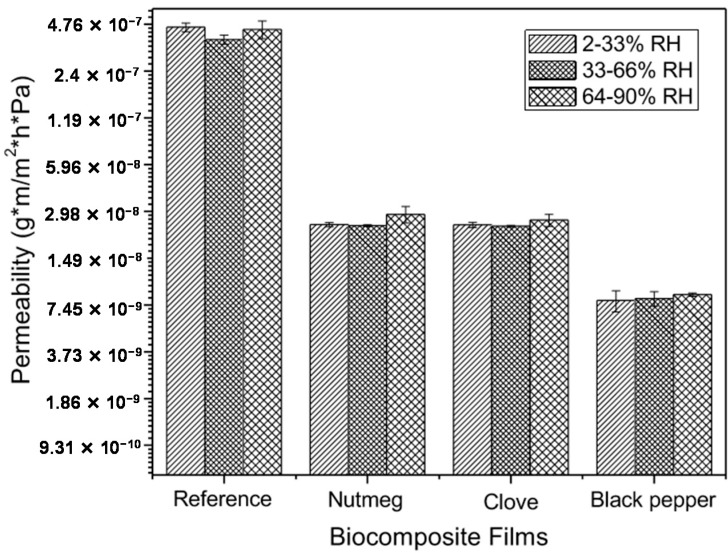
Water vapor permeability of biocomposite films containing oleoresins and reference (without oleoresin). Different letters in the same column indicate significant differences (*p* < 0.05).

Upon adding nutmeg, clove, and black pepper oleoresins to the gelatin matrix, microcrystalline cellulose and glycerol (reference film), the permeability gradient diminished significantly, from 4 × 10^−7^ g·m/m^2^·h·Pa to 4 × 10^−9^ g·m/m^2^·h·Pa (Figure 4). Likewise, upon varying the relative humidity, slight permeability changes were observed among the same films. This permeability decrease with respect to reference films is due to the hydrophobic nature of the oleoresins, which avoids absorption and desorption of water molecules, increasing film hydrophobicity due to the interactions between the hydrophobic compounds in oleoresins and the other film components, which diminishes the availability of the hydrophilic groups. The water molecules diffuse into the continuous polymer phase, where the presence of oily molecules introduces interruptions that increase the tortuosity for transference or mobility of water molecules [46].

#### 3.2.3. Mechanical Properties

**Figure 5:**
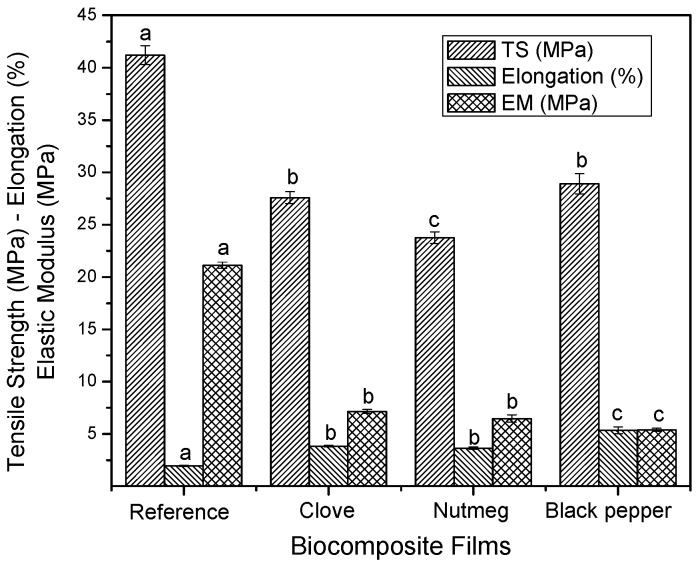
Mechanical properties of biocomposite films with oleoresins and reference. Different letters in the same column indicate significant differences (*p* < 0.05).

The films without oleoresin (reference) were more rigid, possibly due to the restricted mobility of the molecules present in the structure, which could limit their manipulation when used to wrap or package foods. In comparison to these films, biocomposites containing oleoresins showed a reduction in tensile strength (TS) and elastic modulus (EM), as well as a significant increase in their elongation values (Figure 5). This typical behavior of plasticized films has been reported in several studies regarding the addition of lipid components to the biocomposite films. This is attributed to the inability of lipids to form continuous and cohesive matrices when incorporated to biocomposite films [47,48]. As noted in Figure 5, when adding black pepper oleoresin, higher elongation values were obtained. Some authors have indicated that the addition of oily substances such as essential oils (added to the matrix) has a plasticizing role, which gives the material greater flexibility [6,49,50]. Thus, the incorporation of black pepper oleoresin to the polymer gelatin matrix broadens the potential use of this material as food packaging.

#### 3.2.4. Oil Permeability

**Figure 6:**
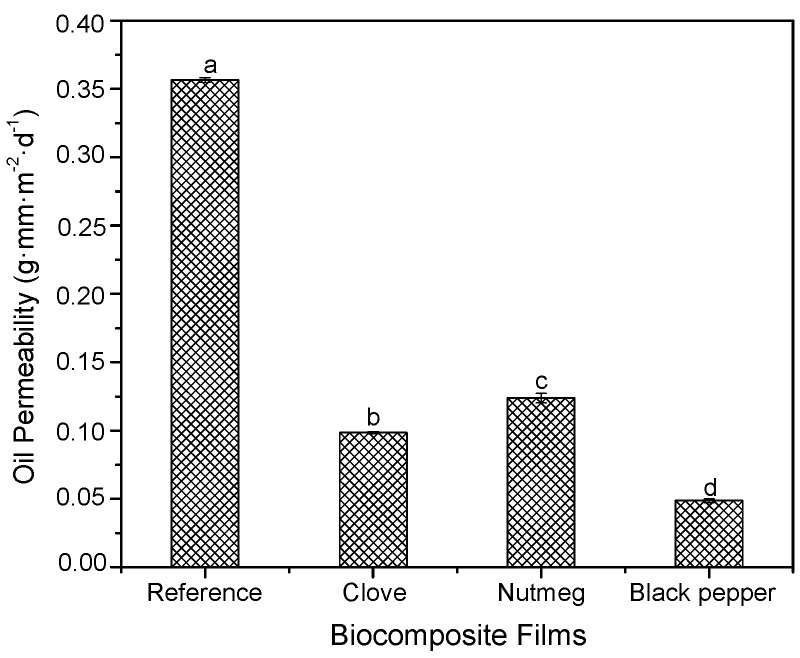
Oil permeability of biocomposite films with oleoresins and reference. Different letters in the same column indicate significant differences (*p* < 0.05).

As shown in Figure 6, oil permeability values of biocomposite films containing oleoresins (clove, nutmeg or black pepper) were between 25% and 30%, smaller in comparison with the reference film (0.36 ± 0.0017 g·mm·m^−2^·d^−1^). This result may be due to a greater final humidity content in films containinig oleoresin (Section 3.2.1) increased the total number of hydroxyl groups (oleophobic) in the film network, which could prevent the passage of oil molecules through the film as reported by [22,51]. Oil permeability values varied significantly in function of the differences in oleoresin composition.

#### 3.2.5. UV-Vis Light Transmittance Values

**Figure 7:**
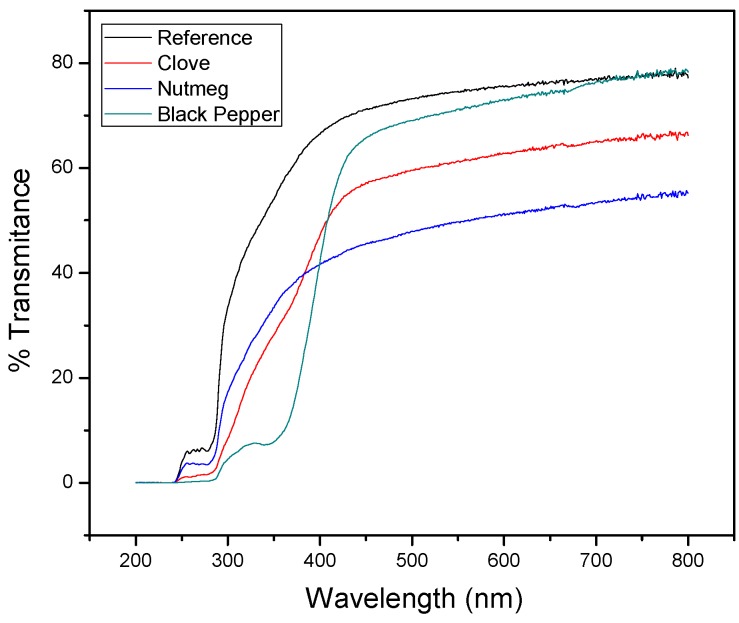
Light transmission of biocomposite films with clove, nutmeg, and black pepper oleoresins and reference (without oleoresin).

As noted in Figure 7, the capacity UV-Vis light transmission of the gelatin films was significantly reduced with the incorporation of oleoresins (*p* < 0.05). This indicated that biocomposite films containing oleoresins (clove, nutmeg or black pepper) enhance their light barrier properties. The reference film had a higher transmittance value at 600 nm (75.39%), followed by the films containing black pepper (73.04%), clove (62.81%), and nutmeg (51.34%) oleoresins. This result could be attributed to light scattering due to the presence of oleoresin in the form of tiny drops dispersed into polymeric matrix. Thus, the light barrier property of the biocomposites is important for food preservation, given that it can avoid photo-oxidation of organic compounds and degradation of vitamins and pigments [9,10].

#### 3.2.6. Measurement of Seal Strength

**Figure 8:**
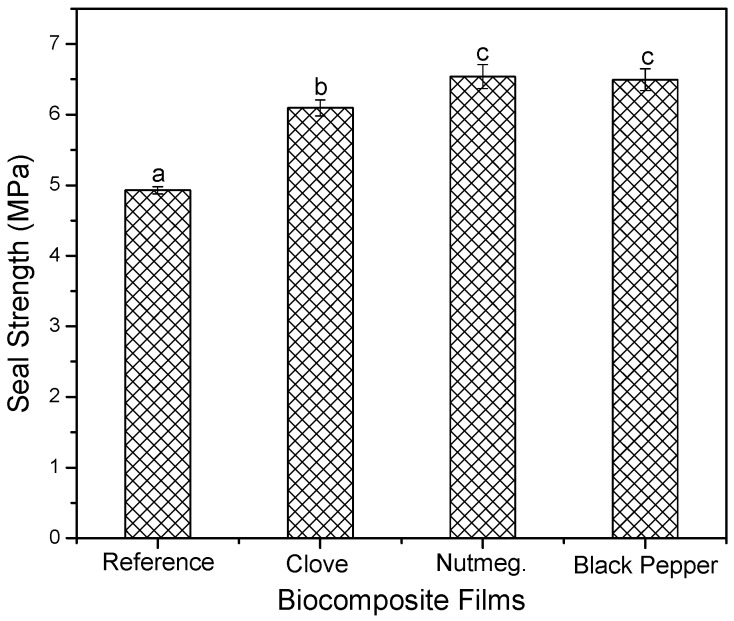
Seal strength of biocomposite films with oleoresins and reference. Different letters in the same column indicate significant differences (*p* < 0.05).

As shown in Figure 8, films containing oleoresins had significant changes with respect to the reference films (without oleoresin) (*p* < 0.05). The reference films had a resistance of 4.93 ± 0.05 MPa. On average, oleoresins increased seal strength by 8% with respect to that of the reference film, achieving greater fusion between the components when heat was applied through the sealer.

#### 3.2.7. X-ray Diffraction (XRD)

**Figure 9:**
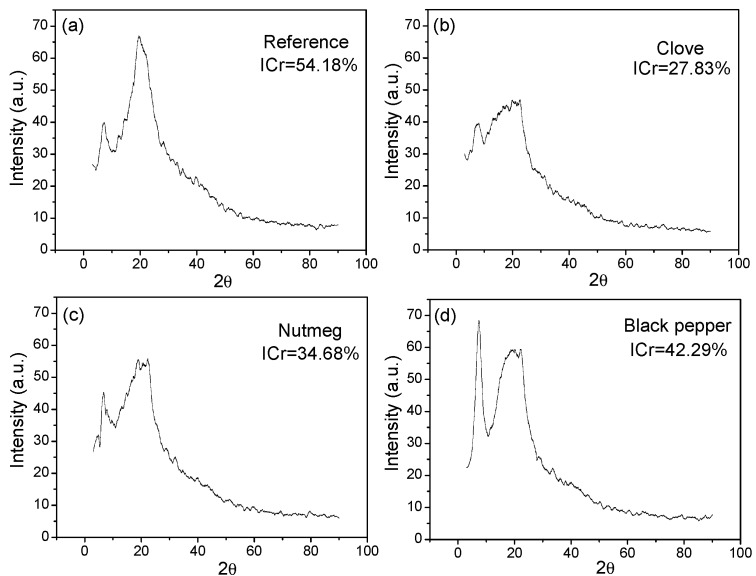
Diffractogram biocomposite films (**a**) Reference (without oleoresin); (**b**) with clove oleoresin; (**c**) with nutmeg oleoresin; (**d**) with black pepper oleoresin.

Figure 9a shows the diffractogram of the reference film, which in the 2θ angle presented two peaks at 19.61° and 7.09°, with 54.18% crystallinity. The diffractogram (Figure 9b), which corresponds to the biocomposite film with clove oleoresin presented two peaks in the 2θ angle at 21.33° and 7.76°, with 27.83% crystallinity. The diffractogram (Figure 9c), corresponding to the biocomposite film with nutmeg oleoresin had two peaks in the 2θ angle at 20.28° and 6.71°, with 34.68% crystallinity. The diffractogram (Figure 9d), corresponding to the biocomposite film with black pepper oleoresin had two peaks in the 2θ angle at 19.95° and 7.09°, with crystallinity of 42.29%. When oleoresins were added to the polymeric matrix, crystallinity diminished with respect to the reference film. This result could be attributed to the fact that the intermolecular interactions among the components of the biocomposite containing oleoresin limited the movements of the molecular chain segments and restrained the crystallisation process [52]. In the present work, the value of the crystallinity obtained for the film containing black pepper oleoresin could indicate good compatibility and interaction with the other components. 

#### 3.2.8. Fourier Transformed Infrared (FTIR) Spectroscopy

**Figure 10:**
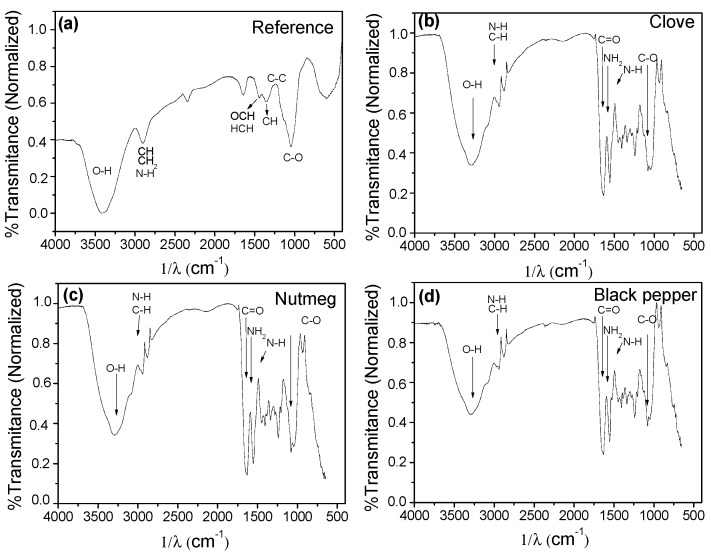
FTIR spectra of biocomposite films. (**a**) Reference (without oleoresin); (**b**) with clove oleoresin; (**c**) with nutmeg oleoresin and (**d**) with black pepper oleoresin.

In the IR spectrum obtained for the reference films (Figure 10a), it can be noted that the band at 3422 cm^−1^ has OH and N-H groups characteristic of glycerol and gelatin (type A amide), respectively. At 2887 cm^−1^, there are CH and CH_2_ groups; the band at 1428 cm^−1^ has the HCH groups and OCH vibrations; the band at 1347 cm^−1^ is related to the vibration of CH groups; the band at 1219 cm^−1^ is attributed to C-C groups; and at 1045 cm^−1^ to C-O group stretching. Most of the functional groups coincide with that reported for microcrystalline cellulose and gelatin. The spectra in Figure 10b–d corresponding to films with clove, nutmeg, and black pepper oleoresins, respectively, were quite similar amongst themselves, but different from the reference film. 

Hydrogen bonds of O-H and N-H groups gave way to a broad band at 3383 and 3199 cm^−1^ because of the compounds in the polymer matrix (gelatin, cellulose, and glycerol); C-H groups were found at 2946 cm^−1^. The band at 1638 cm^−1^ is attributed to C=O bonds; at 1597 and 1479 cm^−1^ NH_2_ primary amines and N-H secondary amines, respectively, and at 1065 cm^−1^ C-O groups. These results indicate that adding oily molecules propitiated a structural change of the polymer matrix material.

#### 3.2.9. Color

The color parameters of the biocomposite films are shown in Table 1. Visually, the films had a slightly yellow aspect and their transparency was reduced upon incorporating the oleoresins. The films containing clove, nutmeg, and black pepper oleoresins had Δ*E* of 4.66, 1.05, and 2.65, respectively, with respect to the reference film.

#### 3.2.10. Thermogravimetric Analysis (TGA)

All the thermograms (not shown) detected four changes of temperature and mass; the first stage revealed a weight loss from 12.73% to 14.37% at temperatures ranging from 150 to 161 °C for all films: This change is related to the loss of free water present in the films and volatile compounds of the oleoresins. On the second stage, the reference biocomposite films and clove films, at temperatures from 273 and 276 °C lost 15.52% and 16.75% mass, respectively; while the films with nutmeg and black pepper at temperatures from 284 and 274 °C lost 20.42% and 20.61% mass, respectively. Within these temperature ranges, degradation occurred of low molecular weight proteins of the polymer (gelatin) and other compounds present in the plasticizer (glycerol). During the third stage, the temperature changes for the biocomposite films ranged between 386 and 402 °C with weight loss between 42.44% and 46.45%, where decomposition took place of high molecular weight proteins present in the gelatin, as well as the degradation of the microcrystalline cellulose due to the rupture of the glycosidic bonds. On the fourth stage, the temperature changes occurred between 598.5 and 598.7 °C for all the films with weight loss between 15.73% and 16.81%, where degradation possibly occurred of the nonvolatile phase of the oleoresins and other compounds of high molecular weight present in the films. 

### 3.3. Physical-Chemical Evaluation of the Bread

#### 3.3.1. Moisture

Table 2 shows that, during the first three days of storage, the bread samples packaged in films containing oleoresins maintained desirable moisture contents according to the norm, while the bread samples without film and those packaged in reference films (without oleoresin) had a significant decrease (*p* < 0.05) in moisture content. After the nine-day storage period, it was noted that the bread samples packaged in films containing black pepper oleoresin achieved a lower loss of moisture compared to the rest, which is desirable to preserve the product’s texture characteristics.

#### 3.3.2. pH

The pH values obtained for all the samples evaluated during the storage period remained within the quality range established by the norm [24]. At nine days of storage, the bread samples packaged in reference films had higher pH values compared to those packaged in films containing oleoresins (Table 2). This result was associated to the presence of acid compounds in the oleoresins. Additionally, bread samples stored without film had the lowest pH values, which could be caused by enzymatic and microbiological reactions catalyzed by the product’s exposure to the environment.

#### 3.3.3. Water Activity (aw)

On the third day of storage, the bread samples packaged in films containing oleoresins maintained a higher water activity as compared to the samples without film and those packaged in reference films. The latter had the lowest values of aw due to the direct exposure of the product to the environment (Table 2). Among the bread samples packaged in films containing oleoresins, those containing black pepper had the highest values of aw. This behavior coincides with the results on humidity content of the bread and permeability to water vapor of said films.

#### 3.3.4. Weight Loss (%wl)

After three days of storage, the bread samples stored in films containing oleoresins had the lowest weight loss values (20–28%). Upon completing the storage period, the bread samples packaged with films containing clove and nutmeg oleoresins had statistically similar weight loss values, while the samples packaged in films containing black pepper oleoresin had the lowest weight loss values (13%). These results confirm the greatest water vapor barrier exerted by the films when incorporating black pepper oleoresin in their formulation.

### 3.4. Microbiological Evaluation of the Bread

Table 3 presents the microbiological quality of bread samples packaged in biocomposite films on days 1, 4, and 9 of storage. Upon completing the storage period evaluated, higher growth of *mold and yeast* occurred on bread samples without film (2440 CFU/g) and in those packaged in reference films (460 CFU/g). Similar behavior occurred in the *S. aureus* count (440 and 50 CFU/g, respectively). In addition, the microbiological analyses evidenced no growth of *S. aureus* and lower growth of *mold and yeast* occurred in the bread samples packaged in films containing oleoresins. During the storage period, it was noted that using biocomposite films containing black pepper oleoresin was the most effective packaging material in terms of the product’s microbiological quality. 

### 3.5. Sensory Analysis of the Bread

**Figure 11:**
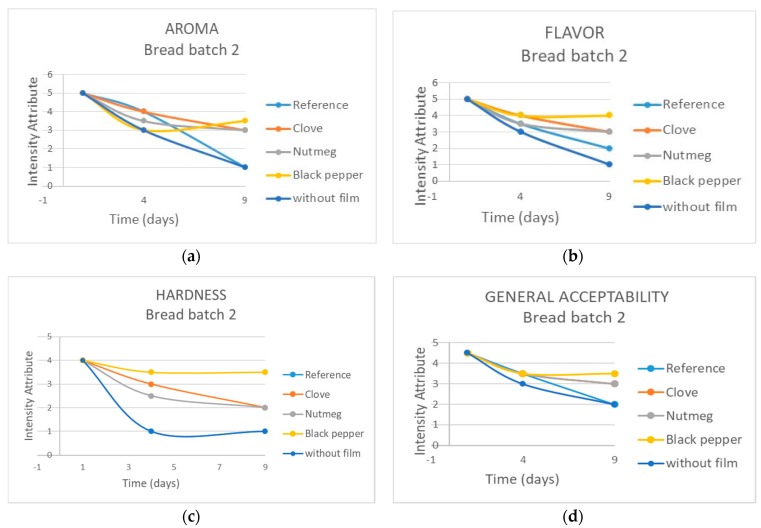
Evolution of sensory attributes during nine days of storage (25 °C and 75% RH) of bread packaged in biocomposite films. (**a**) Aroma; (**b**) flavor; (**c**) hardness; (**d**) general acceptability.

Figure 11a displays the evolution of the aroma attribute of bread in Lot 2 during storage time. Note that on day 1, the maximum score was obtained (I like it a lot) for all the bread samples. On day 4, perceptions were different where the highest score was obtained by the sample packaged in the film with clove and reference (I like it) and the lowest scores were obtained by the samples packaged in films with black pepper and without film (I neither like or dislike it). Figure 11b presents the flavor evolution of the bread packaged in biocomposite films through nine days of storage. It was observed that on day 1 the samples had the same score (I like it a lot); on day 4, the samples packaged in films with clove and black pepper were better perceived (I like it) than those packaged in films with nutmeg, reference, and without film (I neither like or dislike it). Figure 11c shows the hardness evolution of the bread packaged in biocomposite films during the storage period evaluated. On day 1, the panelists assigned the same score to all the bread samples (soft). On day 4, the samples packaged in films with black pepper were scored as soft; the samples packaged in films with nutmeg and clove were perceived as firm and the bread samples packaged in reference films and without film were perceived as very hard. The general acceptability of the bread packaged in biocomposite films is shown in Figure 11d. The sample with the highest acceptability during the storage period was that packaged in films containing black pepper, followed by those packaged in films containing clove or nutmeg. The samples with the best acceptability were those packaged in reference films and without film.

## 4. Conclusions

This study showed that clove, nutmeg, and black pepper oleoresins inhibited *S. aureus* and *E. coli*. Black pepper oleoresin inhibited both strains evaluated, at the lowest concentration studied (0.5 g/100 g of film-forming solution). Incorporation of the aforementioned oleoresins into biocomposite films based on gelatin and microcrystalline cellulose managed to decrease the oil and water vapor permeability values by 20 to 30%. Furthermore, the elasticity and seal strength of the polymeric matrix were increased by 5%. Upon exploring the potential use of films containing the different oleoresins, it was evident that the incorporation of black pepper oleoresin was the most effective packaging material for maintaining the physical-chemical, microbiological, and sensory quality of bread without adding preservatives during nine days of storage.

## Figures and Tables

**Table 1:** CIELab color parameters obtained for biocomposite films.

Biocomposite Films	Color Parameters			
	L *	a *	b *	ΔE *
Reference	92.09 ± 0.094 ^a^	−1.46 ± 0.029 ^a^	4.73 ± 0.33 ^a^	---
Clove	89.59 ± 0.22 ^b^	−1.16 ± 0.039 ^a^	8.65 ± 0.47 ^b^	4.66 ± 0.51 ^a^
Nutmeg	91.30 ± 0.099 ^a^	−1.42 ± 0.014 ^a^	5.44 ± 0.12 ^a^	1.05 ± 0.15 ^b^
Black pepper	90.45 ± 0.77 ^b^	−1.91 ± 0.10 ^a^	6.73 ± 0.79 ^c^	2.65 ± 0.99 ^c^

Reported values correspond to the mean ± standard deviation. Different letters in the same column indicate significant differences (*p* < 0.05). ΔE * calculated on the basis of the L *, a *, b * values of the reference film.

**Table 2:** Physical-chemical characteristics of bread packaged in biocomposite films and stored during nine days at 25 °C and 75% RH.

Bread Packed in Biocomposite Films	Humidity				pH				aw				% Weight loss		
	Day 1	Day 3	Day 5	Day 9	Day 1	Day 3	Day 5	Day 9	Day 1	Day 3	Day 5	Day 9	Day 3	Day 5	Day 9
Reference	26.34 ± 0.79 ^a^	17.94 ± 0.55 ^a^	9.61 ± 0.29 ^a^	7.11 ± 0.2 ^a^	5.73 ± 0.03 ^a^	5.71 ± 0.02 ^a^	5.67 ± 0.02 ^a^	5.64 ± 0.02 ^a^	0.84 ± 0.006 ^a^	0.75 ± 0.006 ^a^	0.58 ± 0.01 ^a^	0.44 ± 0.01 ^a^	45 ± 1.4 ^a^	54 ± 1.5 ^a^	34 ± 1.5 ^a^
Clove	25.69 ± 0.95 ^b^	21.09 ± 0.21 ^b^	11.61 ± 0.9 ^b^	8.86 ± 0.39 ^b^	5.73 ± 0.03 ^a^	5.65 ± 0.02 ^a^	5.63 ± 0.02 ^a^	5.59 ± 0.01 ^a^	0.84 ± 0.006 ^a^	0.79 ± 0.005 ^a^	0.63 ± 0 ^a^	0.54 ± 0.006 ^b^	27 ± 1.3 ^b^	29 ± 1.5 ^b^	17 ± 0.9 ^b^
Nutmeg	26.00 ± 0.55 ^a^	21.05 ± 0.53 ^b^	12.47 ± 0.9 ^c^	8.55 ± 0.59 ^b^	5.73 ± 0.03 ^a^	5.63 ± 0.02 ^a^	5.61 ± 0.02 ^a^	5.57 ± 0.02 ^a^	0.84 ± 0.006 ^a^	0.79 ± 0.01 ^a^	0.67 ± 0.047 ^a^	0.51 ± 0.01 ^a^	28 ± 1.5 ^b^	32 ± 1.5 ^b^	23 ± 1.7 ^b^
Black pepper	25.33 ± 0.49 ^b^	22.21 ± 0.85 ^c^	11.37 ± 0.67 ^b^	9.77 ± 0.45 ^c^	5.73 ± 0.03 ^a^	5.64 ± 0.02 ^a^	5.62 ± 0.02 ^a^	5.57 ± 0.02 ^a^	0.84 ± 0.006 ^a^	0.8 ± 0.01 ^a^	0.67 ± 0.01 ^a^	0.59 ± 0.006 ^b^	20 ± 0.8 ^c^	17 ± 0.9 ^c^	13 ± 1.3 ^c^
Without film	25.59 ± 0.3 ^b^	8.45 ± 0.69 ^d^	5.87 ± 0.25 ^d^	4.71 ± 0.81 ^d^	5.73 ± 0.03 ^a^	5.59 ± 0.02 ^a^	5.56 ± 0.01 ^a^	5.55 ± 0.04 ^a^	0.84 ± 0.006 ^a^	0.52 ± 0.01 ^b^	0.52 ± 0.006 ^b^	0.4 ± 0.01 ^c^	69 ± 1.8 ^d^	41 ± 1.5 ^d^	24 ± 1.8 ^b^

Reported values correspond to the mean ± standard deviation. Different letters in the same column indicate significant differences (*p* < 0.05).

**Table 3:** Microbiological analysis of the bread packaged in biocomposite films and stored during nine days at 25 °C and 75% RH.

Bread Packed in Biocomposite Films	Count *S. aureus* (CFU/g) *			Count Molds and Yeasts (CFU/g) *		
	Day 1	Day 4	Day 9	Day 1	Day 4	Day 9
Reference	<10	<10	50	<10	100	460
Clove	<10	<10	<10	<10	10	50
Nutmeg	<10	<10	<10	<10	10	40
Black pepper	<10	<10	<10	<10	<10	20
Without film	<10	<10	440	<10	80	2440

* Colony-forming units per gram of sample.
